# Development and application of explainable artificial intelligence using machine learning classification for long-term facial nerve function after vestibular schwannoma surgery

**DOI:** 10.1007/s11060-024-04844-7

**Published:** 2024-10-11

**Authors:** Lukasz Przepiorka, Sławomir Kujawski, Katarzyna Wójtowicz, Edyta Maj, Andrzej Marchel, Przemysław Kunert

**Affiliations:** 1 Department of Neurosurgery, Medical University of Warsaw, Banacha St. 1a, 02-097 Warsaw, Poland; 2 Department of Exercise Physiology and Functional Anatomy, Ludwik Rydygier Collegium Medicum in Bydgoszcz Nicolaus Copernicus University in Toruń, Świętojańska 20, 85-077 Bydgoszcz, Poland; 3 Second Department of Radiology, Medical University of Warsaw, Banacha St. 1a, 02-097 Warsaw, Poland

**Keywords:** Machine learning (ML), Artificial intelligence (AI), XAI (eXplainable artificial intelligence), Facial nerve (FN), Vestibular schwannoma, Surgery

## Abstract

**Purpose:**

Vestibular schwannomas (VSs) represent the most common cerebellopontine angle tumors, posing a challenge in preserving facial nerve (FN) function during surgery. We employed the Extreme Gradient Boosting machine learning classifier to predict long-term FN outcomes (classified as House-Brackmann grades 1–2 for good outcomes and 3–6 for bad outcomes) after VS surgery.

**Methods:**

In a retrospective analysis of 256 patients, comprehensive pre-, intra-, and post-operative factors were examined. We applied the machine learning (ML) classifier Extreme Gradient Boosting (XGBoost) for the following binary classification: long-term good and bad FN outcome after VS surgery To enhance the interpretability of our model, we utilized an explainable artificial intelligence approach.

**Results:**

Short-term FN function (tau = 0.6) correlated with long-term FN function. The model exhibited an average accuracy of 0.83, a ROC AUC score of 0.91, and Matthew’s correlation coefficient score of 0.62. The most influential feature, identified through SHapley Additive exPlanations (SHAP), was short-term FN function. Conversely, large tumor volume and absence of preoperative auditory brainstem responses were associated with unfavorable outcomes.

**Conclusions:**

We introduce an effective ML model for classifying long-term FN outcomes following VS surgery. Short-term FN function was identified as the key predictor of long-term function. This model’s excellent ability to differentiate bad and good outcomes makes it useful for evaluating patients and providing recommendations regarding FN dysfunction management.

**Supplementary Information:**

The online version contains supplementary material available at 10.1007/s11060-024-04844-7.

## Introduction

Vestibular schwannomas (VSs) compromise the most common cerebellopontine angle tumors, often detected in middle–aged adults of both sexes with a similar ratio [1, 2]. Although benign, because of a classic triad of hearing loss, tinnitus, and balance disorders, VSs reduce quality of life [3, 4].

Preserving facial nerve (FN) function is a major surgical challenge due to its proximity, compression, and adherence to the tumor [5]. Patients suffering from FN dysfunction face a multitude of challenges, including aesthetic imperfections, psychological distress, and sociological difficulties, among others [6, 7]. This includes abnormal taste sensation as well as dysfunctional lacrimation [8]. All this sums up to a reduced quality of life for patients with FN dysfunction.

In the current study, we applied the machine learning (ML) classifier Extreme Gradient Boosting (XGBoost) for the following binary classification: long-term good and bad FN outcomes after VS surgery. The process of hyperparameter tuning was automated [9]. Because ML models are sometimes described as “black boxes” due to potential limitations in their interpretability [10], we applied an explainable artificial intelligence (XAI) approach to overcome this. The rationale for this is to produce a useful tool for neurosurgeons that may provide explainable results.

ML has been used in previous studies describing VS treatment outcomes [11–15]. Nevertheless, the current study is — according to our best knowledge — the first that applied a ML classifier with an automated method of tuning hyperparameters and providing explanations both at the global and local levels for the evaluation of long-term FN outcomes. The aim of our study is to apply and evaluate these methods on a large, single-center group of VSs within the context of existing findings from prior studies.

## Methods

The Bioethics Committee of the Medical University of Warsaw approved the study’s protocol and waived patient consent due to the study’s retrospective design and participants’ anonymity. We retrospectively evaluated patients operated on for sporadic VS between the years 2000 and 2016. Patients diagnosed with neurofibromatosis type 2 were not included in this study. Two patients were excluded due to death, nine had no follow-up and were not included either. As a result, a group of 256 consecutive patients was analyzed.

The group consisted of 98 men (38.5%) and 158 women (61.5%) with a median age of 49 (Q1 = 38, Q3 = 58). Median tumor size was 2.9 cm (Q1 = 2.1, Q3 = 3.6), median tumor volume was 8.2 cm^3^ (Q1 = 3.6, Q3 = 18.6), and a retrosigmoid approach was used most (253, 98.8%), with 4 patients (1.1%) undergoing a translabyrinthian approach, while the remaining 2 patients (0.6%) had both. Surgeries were performed by 5 neurosurgeons, of which the senior author (AM) performed the majority (219, 84.9%). The median observation period was 3 years (range: 0.5 – 12 years), and only 6 patients had an observation period under 1 year. Extent of tumor resection, confirmed with follow-up MRI, is available for 249 patients. Of these, 241 had gross total resections (GTRs) (96.8%), 1 had partial resection and remained stable (0.4%), 2 underwent revision translabyrinthine resection (0.8%), 1 underwent postoperative radiosurgery (0.4%), 2 are under observation for potential regrowth (0.8%), and 2 had GTRs with postoperative CT scans due to contraindications for MRI (0.8%).

Other examined factors are listed in Table 1. The postoperative complications included: cerebrospinal fluid leak (n = 21), lower cranial nerve deficits (n = 19), cerebellar symptoms (n = 13), hematoma at the resection cavity (n = 6), cerebellar edema (n = 3), pneumocephalus (n = 2), meningitis (n = 2), pneumonia (n = 1), C. difficilie infection (n = 1).

**Table 1:** Group comparison

Characteristic	Bad outcome (n = 86, 34%)	Good outcome (n = 170, 66%)	p–value	q–value
Age during the surgery years	50 (39, 59)	49 (37, 57)	0.24	0.30
Sex			0.006	0.025
female	43 (50%)	115 (68%)		
male	43 (50%)	55 (32%)		
Year of surgery	2,008 (2,005, 2,013)	2,010 (2,006, 2,013)	0.12	0.19
First symptom			0.17	0.23
incidental finding	0 (0%)	2 (1.2%)		
deafness	4 (4.7%)	2 (1.2%)		
hearing loss	51 (59%)	74 (44%)		
tinnitus	18 (21%)	58 (34%)		
ear pain	0 (0%)	2 (1.2%)		
dizziness	2 (2.3%)	5 (2.9%)		
headache	5 (5.8%)	12 (7.1%)		
trigeminal nerve signs	2 (2.3%)	9 (5.3%)		
facial nerve paresis	0 (0%)	1 (0.6%)		
trigeminal neuralgia	0 (0%)	1 (0.6%)		
cerebellar signs	3 (3.5%)	3 (1.8%)		
papilledema	1 (1.2%)	1 (0.6%)		
Reason for the diagnosis				
incidental	6 (7.0%)	5 (2.9%)		
deafness	8 (9.3%)	12 (7.1%)		
hearing loss	27 (31%)	68 (40%)		
tinnitus	7 (8.1%)	18 (11%)		
dizziness	6 (7.0%)	16 (9.4%)		
headache	5 (5.8%)	14 (8.2%)		
trigeminal nerve signs	7 (8.1%)	18 (11%)		
facial nerve paresis	1 (1.2%)	2 (1.2%)		
lower cranial nerve paresis	1 (1.2%)	0 (0%)		
cerebellar signs	13 (15%)	16 (9.4%)		
visual symptoms	5 (5.8%)	1 (0.6%)		
Tinnitus			0.017	0.044
no	50 (58%)	72 (42%)		
yes	36 (42%)	98 (58%)		
Hearing loss			0.019	0.047
no	2 (2.3%)	11 (6.5%)		
yes	60 (70%)	135 (79%)		
bilateral	24 (28%)	24 (14%)		
Dizziness			0.64	0.66
no	67 (78%)	128 (75%)		
yes	19 (22%)	42 (25%)		
Cerebellar signs			0.032	0.067
no	62 (72%)	142 (84%)		
yes	24 (28%)	28 (16%)		
Trigeminal nerve signs			0.11	0.18
no	50 (58%)	116 (68%)		
yes	36 (42%)	54 (32%)		
Preoperative headache			0.18	0.23
no	70 (81%)	149 (88%)		
yes	16 (19%)	21 (12%)		
Preoperative hydrocephalus			> 0.99	> 0.99
no	85 (99%)	167 (98%)		
yes	1 (1.2%)	3 (1.8%)		
Previous treatment history			0.006	0.025
no	80 (93%)	169 (99%)		
yes	6 (7.0%)	1 (0.6%)		
Duration of symptoms months	30 (12, 60)	24 (10, 58)	0.14	0.20
Preoperative ABR			0.001	0.006
normal	1 (1.2%)	2 (1.2%)		
not tested	20 (23%)	57 (34%)		
abnormal	20 (23%)	64 (38%)		
no response	45 (52%)	47 (28%)		
Hannover hearing classification			0.017	0.044
1	5 (5.8%)	1 (0.6%)		
2	2 (2.3%)	12 (7.1%)		
3	13 (15%)	32 (19%)		
4	8 (9.3%)	28 (16%)		
5	58 (67%)	97 (57%)		
Preoperative House–Brackmann scale			0.007	0.025
1	62 (72%)	147 (86%)		
2	18 (21%)	20 (12%)		
3	2 (2.3%)	3 (1.8%)		
4	1 (1.2%)	0 (0%)		
6	3 (3.5%)	0 (0%)		
Samii classification of vestibular schwannoma extension			< 0.001	< 0.001
T1	1 (1.2%)	0 (0%)		
T2	1 (1.2%)	8 (4.7%)		
T3a	3 (3.5%)	32 (19%)		
T3b	4 (4.7%)	30 (18%)		
T4a	21 (24%)	42 (25%)		
T4b	56 (65%)	58 (34%)		
Tumor linear size, cm	3.5 (2.8, 4)	2.6 (1.9, 3.3)	< 0.001	< 0.001
Tumor volume, cm3	14 (7, 22)	6 (3, 13)	< 0.001	< 0.001
Side of the tumor			0.078	0.14
left	36 (42%)	91 (54%)		
range	50 (58%)	79 (46%)		
Internal auditory canal widening			0.29	0.33
normal	6 (7.0%)	19 (11%)		
widened	80 (93%)	151 (89%)		
Surgeon			0.040	0.081
Surgeon 1 (AM)	79 (92%)	140 (82%)		
Surgeon 2	1 (1.2%)	9 (5.3%)		
Surgeon 3	2 (2.3%)	1 (0.6%)		
Surgeon 4 (PK)	4 (4.7%)	20 (12%)		
Surgical approach retrosigmoid			0.26	0.32
no	2 (2.3%)	1 (0.6%)		
yes	84 (98%)	169 (99%)		
Surgical approach translabyrinthine and retrosigmoid			0.34	0.37
no	85 (99%)	170 (100%)		
yes	1 (1.2%)	0 (0%)		
Tumor structure			0.13	0.19
cystic	21 (24%)	28 (16%)		
solid	65 (76%)	142 (84%)		
Surgical position			0.013	0.041
lateral decubitus	25 (29%)	27 (16%)		
supine with head turned	61 (71%)	143 (84%)		
Neuromonitoring			0.34	0.37
no	1 (1.2%)	0 (0%)		
yes	85 (99%)	170 (100%)		
Facial nerve dislocation pattern			0.081	0.14
anterior and superior to the tumor	35 (41%)	81 (48%)		
anterior to the tumor	13 (15%)	34 (20%)		
superior to the tumor	26 (30%)	44 (26%)		
posterior	0 (0%)	1 (0.6%)		
inside the tumor	3 (3.5%)	0 (0%)		
no data	9 (10%)	10 (5.9%)		
Perioperative use of nimodipine			0.077	0.14
no	49 (57%)	77 (45%)		
yes	37 (43%)	93 (55%)		
Intraoperative difficulties			< 0.001	< 0.001
none	61 (71%)	155 (91%)		
adhesions	11 (13%)	7 (4.1%)		
excessive bleeding	14 (16%)	8 (4.7%)		
Histopathology			0.53	0.56
Antoni A	19 (22%)	48 (28%)		
Antoni B	8 (9.3%)	17 (10%)		
Antoni A and B	59 (69%)	105 (62%)		
Preserved hearing after the surgery			0.012	0.041
hearing loss	3 (3.5%)	23 (14%)		
deafness	83 (97%)	147 (86%)		
Postoperative complications			0.021	0.049
no	60 (70%)	140 (82%)		
yes	26 (30%)	30 (18%)		
Short-term facial nerve function			< 0.001	< 0.001
1	0 (0%)	27 (16%)		
2	0 (0%)	40 (24%)		
3	4 (4.7%)	47 (28%)		
4	31 (36%)	50 (29%)		
5	23 (27%)	6 (3.5%)		
6	28 (33%)	0 (0%)		
Long-term facial nerve function				
1	0	80 (47.1%)		
2	0	90 (52.9%)		
3	33 (38.4%)	0		
4	16 (18.6%)	0		
5	11 (12.8%)	0		
6	26 (30.2%)	0		

Values provided are median (IQR) for numeric features or n (%) for qualitative features

q-value and p-value after false discovery rate correction. Values are not provided for reason for the diagnosis and long-term facial nerve function

To allow statistical evaluation of binary outcomes, House-Brackmann grades 1–2 were considered to be a good long–term result of FN function, while grades 3–6 denoted a bad outcome. This choice was arbitrary (as opposed to the 1–3 and 4–6 division), but it helped avoid further class imbalance and aligned with similar studies in the literature [16–19]. Boosting techniques are typically more effective in binary classifications and can be further leveraged by converting ordinal objectives into binary ones. Additionally, this method improves interpretability of the model classifications [20].

### Statistical analysis

Descriptive statistics were performed using JAMOVI [21]. Continuous variables were compared between groups with good and bad outcomes after surgery using the U-Mann Whitney test, while categorical variables were compared using Pearson’s Chi-squared test, or Fisher’s exact test. Q-values are provided as a false discovery rate correction for multiple testing. The programming work for machine learning was performed in the Python programming language (version 3.9) [22]. All data pre-processing and analysis were done using Pandas [23] and NumPy [24]. Scikit-learn [25] was used to prepare evaluation metrics and a confusion matrix. Highly correlated and redundant variables were removed.

The obtained dataset consisted of a binary target variable, 34 predictors (features), and 256 rows (patients). Character variables were coded as integers. Five-folds cross-validation (CV) was applied to obtain the following classification average performance scores: area under the curve (AUC), receiver operating character (ROC), classification accuracy (CA), F1, precision, recall, and Matthew’s correlation coefficient (MCC). The likelihood that a randomly selected positive example (good outcome) would be scored higher by the classifier than a randomly selected negative example (bad outcome) is represented by the AUC [26]. It has a range of 0.5 for random guessing and 1.0 for perfect categorization. AUC values greater than 0.8 are often regarded as clinically meaningful [27]. CA is the ratio of correctly predicted instances (outcomes) to the total number of instances (outcomes). However, this metric can be misleading in imbalanced datasets. The ratio of true positives to the sum of false positives and true positives (precision) reflects the accuracy of a classifier. It indicates how many predicted positive cases actually turn out to be positive. The ratio of true positives to the total of true positives and false negatives, is often referred to as sensitivity or true positive rate or recall. It gauges how many true positive cases (good outcomes) were accurately detected [28]. The harmonic mean of recall and accuracy is known as the F1-score. The value representing the best performance is 1, and the range is 0–1 [29]. The MCC accounts for both true and false positives and negatives, and therefore is regarded as a balanced metric. It has a range of − 1 to 1, where 1 denotes an exact classifier, 0 is a random prediction, and − 1 is a complete discrepancy between the prediction and the observation. MCC seems to be especially helpful for unbalanced datasets [28].

Analysis of the classification of good vs. bad outcomes was applied with a ML method using the XGBoost package for Python [30]. The model choice was based on the existence of a variety of packages useful in training, analysis, and interpretation of results. The application of these packages might serve for the creation of a background for a semi-automatic pipeline for the prediction of long-term FN function after VS surgery. Optuna package was used to optimize hyperparameters [9]. Within the package, following hyperparameters were set as fixed: the objective was chosen as binary: logistic, seed = 42, the tree method was chosen as” hist”, and booster was chosen as: “gbtree”, grow policy was selected as default (“depthwise”), “eval_metric”: “auc”, and scale_pos_weight as 1.98, The mechanism for building trees is specified by the tree_method option. The “hist” approach is a fast algorithm that accelerates training, by utilizing histogram-based techniques. The measure that is used to assess the model’s performance during training is specified by the eval_metric parameter. For binary classification problems, AUC is a widely used statistic that assesses the model’s ability to discriminate between classes. “gbtree” denotes the typical XGBoost usage of decision trees as base learners in the model. The dataset’s class imbalance (170 good outcomes (negative instances) vs 86 bad outcomes (positive instances) is addressed with the scale_pos_weight option. The following hyperparameters were subjected to tuning based on the results of the 1000 trials using Optuna [9]. Reg_alpha and reg_lambda were chosen from values between 0 and 1, with a step of 0.1. Gamma was chosen from values between 0 and 5, with a step of 0.2. Subsample and colsample_bytree were chosen from values from 0.5 to 1.0 with a step of = 0.1. Max_depth was chosen from values from 3 to 10, with a step of 1. Min_child_weight was chosen from values from 1 to 10, with a step of 1. Eta (learning rate) was chosen from values from 0.01 to 0.3 with a step = 0.1. N_estimators were chosen from values 100 to 500, with step = 10. Eventually, the following values were chosen based on the best Optuna study trial: eta = 0.21, max_depth = 3, n_estimators = 390, gamma = 2.8, reg_alpha = 0.1, reg_lambda = 1, colsample_bytree = 0.9, subsample = 0.6, min_child_weight = 1. As the XGBoost algorithm approaches the loss function’s minimum, the eta parameter—also referred to as the learning rate—manages the step size at each iteration. In terms of balancing the danger of overfitting the ideal solution with the speed of convergence, a value of 0.21 could be considered moderate. The maximum depth of the decision trees is specified by the max_depth option. A shallow tree structure (represented by a value of 3) helps control the complexity of the model, which helps prevent overfitting. The number of boosting rounds or trees to be constructed is indicated by the n_estimators parameter. A value of 390 indicates use of several trees to enhance prediction accuracy. Gamma parameter, or the lowest loss reduction required to make a split, helps control the complexity of the model. With a value of 2.8, the model is more cautious because a split will only take place if it significantly lowers the loss. The weights undergo L1 regularization (Lasso) via the reg_alpha parameter. A value of 0.1 encourages sparsity in the model by introducing a penalty for large coefficients. By penalizing large coefficients, the reg_lambda parameter smoothes the model by applying L2 regularization (Ridge) on the weights. A high amount of regularization, with a value of 1, can help prevent overfitting. The percentage of features (predictors) to be randomly selected for each tree is specified by the colsample_bytree option. Ninety percent of the features (predictors) are used to create each tree when the value is 0.9, which increases variation among the trees and decreases overfitting. The percentage of observations that will be used to develop each tree is indicated by the subsample parameter. Introducing randomization into the training process helps to prevent overfitting; a value of 0.6 means that 60% of the observations is picked randomly. The minimal total instance weight (hessian) required in a child is indicated by the min_child_weight option. Setting min_child_weight = 1 can help handle this by preventing the algorithm from creating nodes with very few instances of the minority class.

The ranking of features and their one–way interaction was done using the xgbfir package. The following metrics were used: gain, which is the total gain of each feature or feature interaction; wFScore which is the number of possible splits taken on a feature or feature interaction weighted by the probability of the splits to take place; Average wFScore which is a wFScore divided by FScore, average Gain which is Gain divided by FScore, and Expected Gain, which is the total gain of each feature or feature interaction weighted by the probability of gathering the gain. [31] To explain the obtained classifications, we applied local interpretable model–agnostic explanations (LIME) and SHapley Additive exPlanations (SHAP) [32, 33]. Both SHAP and LIME could be used to explain the prediction of the ML model for a specific patient, and — accordingly — could provide local explanations and interpretations [34, 35]. In addition, SHAP also provides global explanations and interpretations by assessing each input variable’s contribution to the model’s overall predictive ability of the model [34]. SHAP and LIME might serve as complementary methods, and both of them have been used in previous studies in medicine. [32, 36] SHAP values were used to explain global model classification [34]. Explanation of features (predictors) using Permutation Importance was calculated based on 10 iterations. The LIME package was used to present a local explanation of the model [35].

## Results

Table 1 presents a comparison between patients with good and bad outcomes after the surgery. Table S1 presents a description of the data used and the information on which numeric values were used to encode categorical classes. Because most of the patients obtained good outcomes (n = 170, 66%, Table 1), there is an imbalance in target (predicted) categories in the overall sample. Figure S1 presents a correlation heatmap of features and the target using the Kendall rank correlation coefficient. Short–term FN function (tau = − 0.6) was the only feature that correlated with long–term FN function. Patients with good outcomes had both smaller tumor volume and smaller linear tumor sizes (6 cm3 vs 14 cm3 and 2.6 cm vs 3.5 cm, respectively, Table 1).

XGboost classifier with tree_method = ‘hist’, and scale_pos_weight = 1.98 performance was compared to other baseline, “vanilla” classifiers: Logistic Regression, Decision Tree, and Random Forest (Table S2). Logistic regression outperforms the XGBoost classifier in 2 from 6 scores in total; nevertheless, the XGBoost classifier was picked as a model of choice, as it is more prone to hyperparameters tuning than Logistic Regression.

The results of 1000 experiments conducted to delineate the most optimal values of hyperparameters are shown in Figure S2. Average model accuracy based on five-fold CV was 0.83, ROC AUC score was 0.91, and the MCC score was 0.62, precision: 0.74, recall: 0.76, and f1: 0.74.

**Fig. 1:**
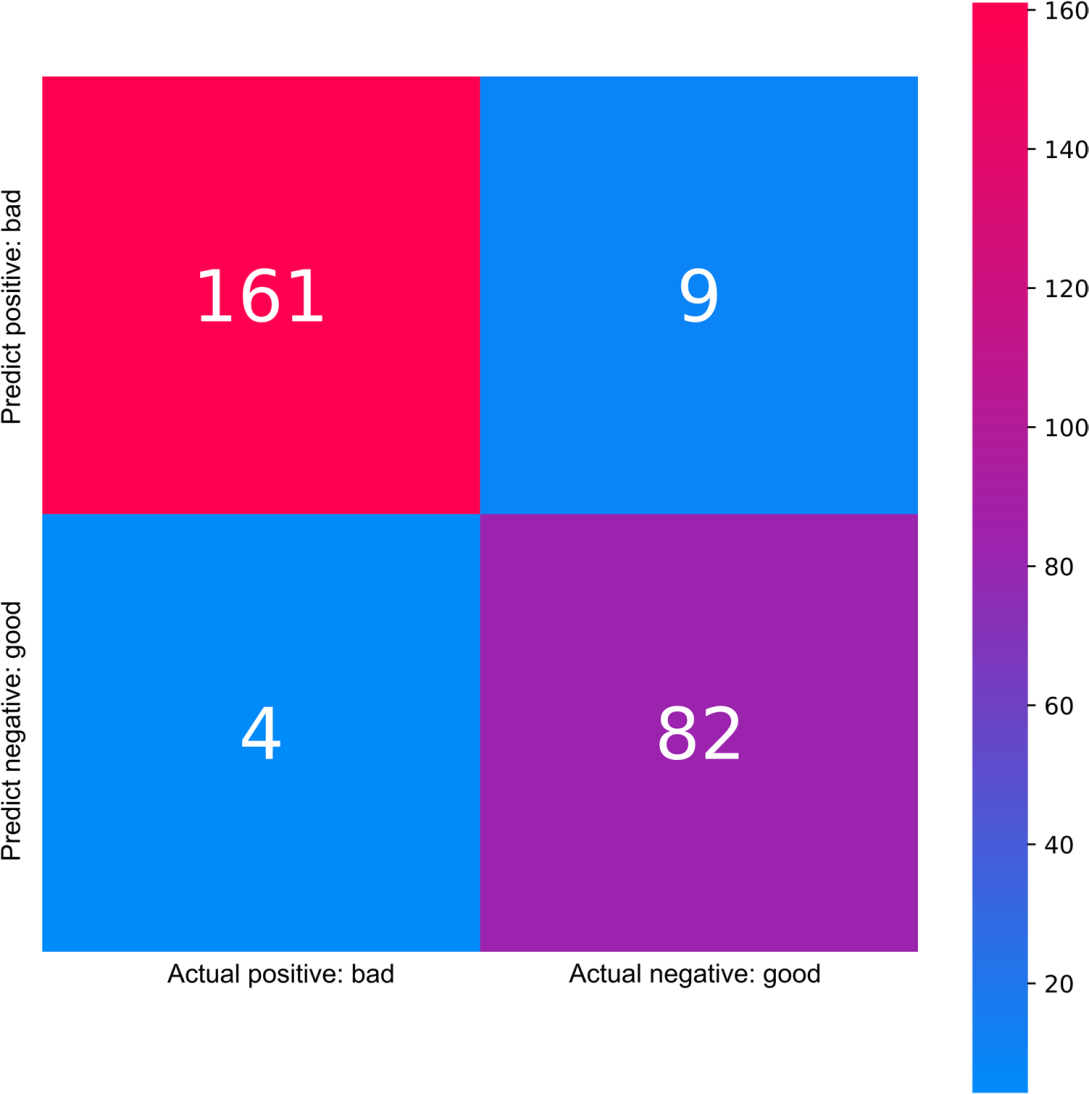
Confusion matrix for the XGBoost classifier

Figure 1 presents a confusion matrix for the XGBoost classifier. Classifications of and bad outcomes were characterized by the presence of both false positives (n = 9) and false negatives (n = 4).

**Fig. 2:**
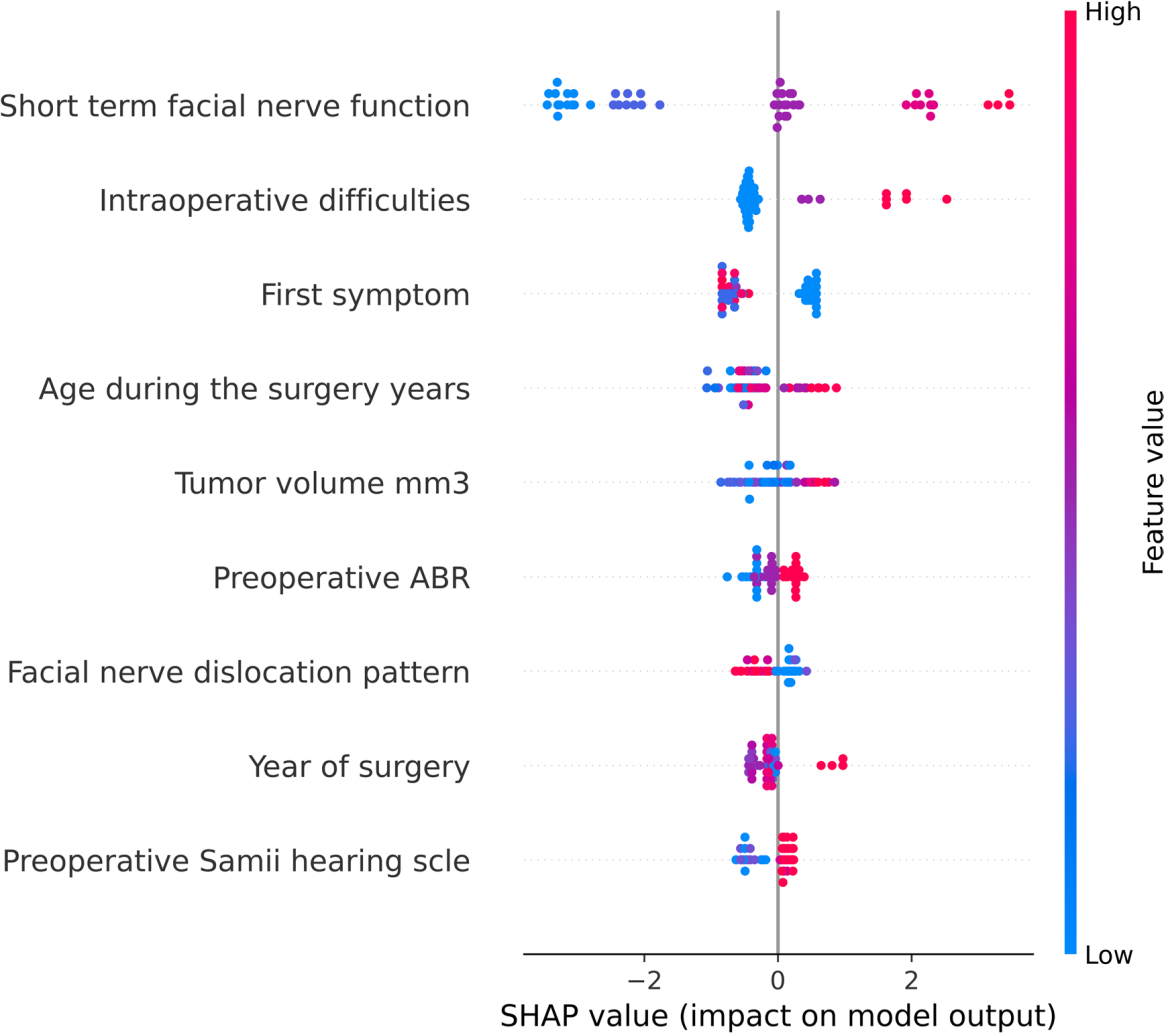
SHapley Additive exPlanations (SHAP) values for the applied classifier predicting a bad outcome

Figure 2 presents SHAP values for the applied classifier predicting bad outcomes. Predictors (features) are ordered from the most important according to SHAP values (top of the figure) to the least important (bottom of the figure). The most important feature according to SHAP values was short-term FN function. High and mid values (red and purple dots respectively) of short term FN function, intraoperative difficulties (such as excessive bleeding, denoted by red dots), first symptoms as incidental finding (denoted as blue dots), higher patient age during the surgery, higher values of tumor volume, no response in preoperative ABR (denoted as red dots), anterior FN dislocation pattern (denoted as blue dots) and higher values of preoperative Samii hearing scale had a high positive contribution to bad outcomes after surgery. The remaining features did not make a high contribution to the model output.

Figure S3 presents a single decision tree based on the XGBoost model. If the value of “short term FN function” is lower than 4, then a decision would be made based on intraoperative difficulties. If there were no excessive bleedings intraoperatively, then the patients would be classified as “good outcome” (Figure S3). If short term FN function would be 4 or higher, then the same feature would be considered with 5 points as a cut-off. If the patient would have a value lower than 5, then preoperative ABR would be taken into account. If this parameter would be of value other than “no response” (denoted as “2”), then the patient would be classified as a “bad outcome”, otherwise as a “good outcome”.

Potential interaction between features (predictors) has been assessed using the xgbfir package. Table S3 shows the top 9 features (depth 0) and interactions of depth 1. Various feature ranking metrics showed that, in general, features are characterized by a higher gain when considered alone than their interaction term (Table S3). Therefore, it can be concluded that those features have no significant interaction in the relationship with a surgical outcome.

Figure S4 shows feature importance using 3 techniques. Panel A (Figure S3A) shows feature importance using the XGBoost in-built algorithm, Figure S4B shows permutation feature importance using the Scikit-learn package, and FigureS4C using SHAP. There are many similarities between results in the delineation of the most important features for the classification. For instance, short–term FN function is the most important feature according to all three methods used (Figure S4).

**Fig. 3:**
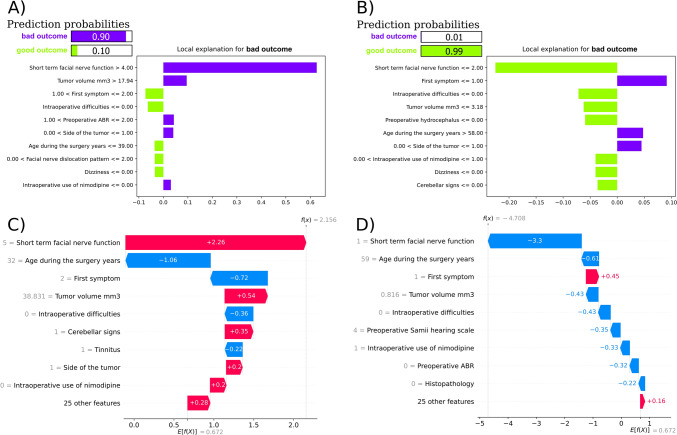
Panel A shows the LIME explanation for a selected patient correctly predicted to have a bad outcome, while panel B shows a good outcome. The green color denotes a negative effect on the prediction of bad outcome after surgery, while purple denotes a positive effect. The black text after the feature names shows the value of each feature for this patient. Panel C shows the SHAP explanation for a selected patient predicted to have a bad outcome, while panel D shows a good outcome presented with a waterfall plot. Red bars denote a positive effect on the prediction of bad outcome after surgery, while blue bars denote a negative effect. The gray text before the feature names shows the value of each feature for this patient

Figure 3 represents a local explanation for two selected instances (patients): one correctly predicted to have a good outcome (right side of the figure, panels B and D) and one with correctly predicted bad outcome (left part of the figure, panels A and C) using LIME (panels A and B) and SHAP (panels C and D). The LIME algorithm correctly assigned a probability of 0.90 and 0.99 of a bad and good outcome after surgery, respectively. The SHAP explanation had a 0.672 expected value in the model output for all instances (patients). For patients with a bad and good outcome, the predicted values were 2.156, and -4.708, respectively. Similarly, to SHAP, the most important predictor according to LIME was short-term FN function. The relationship with the target is similar as in the case of SHAP values, i.e., high values (House-Brackmann grade 5) were related to bad outcome, while low values (House-Brackmann grade 1) were related to good outcome. In addition, according to LIME and SHAP, high volume of tumor (17.94 cm3 and higher according to LIME) was related to classification as bad outcome (Fig. 3 panels A and C). Hearing loss as the first symptom was related to bad outcome in patient with correctly predicted good outcome (Fig. 3 panels B and D).

## Discussion

In this study, we developed and employed XAI based on the results of ML classification for long-term FN function evaluation after VS surgery. Thanks to the application of an XAI approach we were able to describe the importance and direction of influence of features on the results. The risk factor that most consistently emerged and was denoted as the most important was short–term FN function. Additional analysis showed that intraoperative excessive bleeding and short-term FN function have a significant interaction between each other in relation to a surgical outcome (Table S3). The correlation between these two features is low (Figure S1).

The most important feature according to SHAP values was short-term FN function. High and mid values of short-term FN, intraoperative difficulties such as excessive bleeding, first symptoms as incidental, greater age during the surgery, higher values of tumor volume, no response in preoperative ABR, anterior FN dislocation pattern, higher values of preoperative Samii hearing scale had a high positive contribution to bad outcomes after surgery. These results could assist in the early identification and close monitoring of patients who may require FN neuroraphy, upper eyelid implants, masseter muscle transfer or other techniques designed to manage FN dysfunction. In other words, this model may allow for a more personalized post-operative care and targeted rehabilitation, eventually leading to improved quality of life.

### Novelty of this study

According to our best knowledge, this is the first applied ML classifier with an automated method of setting up hyperparameters and providing explanations at both the global and local levels (XGBoost with optuna, LIME, and SHAP). In addition, a tree has been visualized to present a mechanism underlying the decision made by the applied classifier. Such an approach allows the use of a partially-automated pipeline for creating, and tuning hyperparameters of a ML algorithm potentially useful in classifying the outcome of long–term FN function after VS surgery.

### Factors predicting long–term facial nerve outcome

Our results are in line with other studies of predictive factors of long–term FN outcomes using traditional statistical methods. Particularly, in prospective studies by Fenton et al. and Ren et al., the researchers found that immediate FN function was a predictor of long-term FN function [17, 37]. Other studies commonly report tumor size among other important predictors of long–term FN outcomes [16, 19, 38–40]. Another noteworthy intraoperative method involves supramaximal stimulation, a technique described by Schmitt et al., which has proven useful in predicting favorable FN function [41].

In the current study, XGBoost classifier predictions were explained using LIME and SHAP. Low House-Brackmann grades at discharge (i.e., good short-term FN function) as well as the relatively small size of the tumor have a positive effect on the prediction of good outcomes after surgery. However, not all local (single patient-level) explanations were identical when comparing SHAP to LIME. This difference might be explained by the fact that even the most important feature in the current study did not perfectly divide groups according to target. It was shown based on one dataset that SHAP appeared to be superior to LIME weights when it comes to explaining the results of the XGBoost classifier, in terms of discriminative power [42]. However, when examined on different types of data, no particular interpretability technique is superior across all performance metrics [43].

The decision tree derived from the XGBoost model introduced in this study encompassed variables that had otherwise limited influence on long-term FN function. Nevertheless, it is crucial to acknowledge that decision trees are not conventionally employed for data interpretation.

### Study limitations and future directions

In this study, bad outcomes were noted in 34% of patients, while good outcomes occurred in 66%, resulting in an imbalanced distribution of target classes. The application of machine learning techniques, such as XGBoost, on imbalanced datasets requires further studies [44]. However, when the minority-to-majority ratio in machine learning is between 1:100 and 1:10,000, there is a significant degree of class inequality [45] which is significantly greater than in the data examined in this research. Additionally, the “scale_pos_weigh” hyperparameter in XGBoost, which was used in the current study, may be successfully tuned to resolve relatively tiny target imbalances like those in the current data [44]. In addition, classification of the applied XGBoost model was characterized by the presence of 9 false positives (4% out of all classifications: nine patients were predicted to have a bad outcome by a model, while the grand truth was a good outcome). Nevertheless, the greatest decrease in classification performance occurs for the distribution with only 5 percent positive classes [46]. The ROC AUC score of the applied model was 0.91, which could be considered excellent [47]. One potential strength of the study is that we also applied the MCC score, which might be superior in comparison to the accuracy, and the F_1_ score in the case of the binary classification task if positive and negative cases are of equal importance [29]. An MCC value of 0 is expected for the trivial majority classifier as well as for the coin-tossing classifier, while a perfect classifier would have a MCC =  + 1 [29]. In the current study, the MCC was 0.62. Further studies on the application of ML models, including XGBoost, on imbalanced datasets are needed [44].

The assumption of feature independence by SHAP and LIME is one of their main limitations. When features are related, false conclusions might be drawn based on these methods. For instance, LIME creates perturbed samples by using the original instance as a basis, which may result in irrational feature value combinations if features are related to each other. As a result, feature significance may be interpreted incorrectly as the local surrogate model trained on these disturbed samples might not correctly reflect the grand truth relationship between the features and the prediction [48]. SHAP encounters comparable difficulties. Its focus on the independence of features can lead to biased interpretations when correlations occur. The chosen machine learning model has a major impact on both approaches [49]. Therefore in the current study, feature selection was based on the collinearity, to exclude redundant features from the dataset. Another significant drawback of these techniques is their computational complexity and potential computational cost, which grows with the number of features. Even though LIME is often faster than SHAP, training a local surrogate model requires producing a significant number of perturbed data. Therefore, this procedure may need a lot of resources, particularly if the underlying model is complicated [48, 50]. Nevertheless, in the current study, analyzed data was rather very sparse in terms of big data norms, and the model was not highly complex, therefore computation power was not a substantial issue in this case.

Although our examined dataset is relatively small from a ML perspective (n = 256 patients), it represents a substantial cohort in the realm of VS surgery, where a significant group of patients has been gathered for analysis. Tabular data is recognized as an”unconquered castle” for “deep” models [51, 52]. Currently, “shallow” models as gradient-boosted decision trees are considered a gold standard method in tabular data applications [53, 54]. In general, it is recognized that increasing sample size also increases model classification performance [46, 55].

Finally, our study is limited by its retrospective and single–institutional nature. Consequently, future studies should evaluate this model in a prospective manner, preferably in a multi–center setting. The identification of preoperative factors that strongly and reliably influence long-term FN function may improve patient counselling, facilitate discussions about treatment goals and expectations, and assist in surgical planning.

## Conclusions

We presented a ML model that proved to be effective in classifying long–term FN outcomes after VS surgery. Short–term FN function emerged as the key feature associated with long–term good outcomes. With its excellent ability to differentiate between bad and good outcomes, the model can be used in clinical practice for evaluating patients and providing treatment recommendations. In particular, it may facilitate early identification and close monitoring of patients who might require additional techniques to manage FN dysfunction. Further studies on AI applications in skull base surgery may consider applying an explainable approach.

## Supplementary Information

Below is the link to the electronic supplementary material.

Supplementary file1 (PNG 5432 KB)—Correlation matrix heatmap

Supplementary file2 (PNG 2152 KB)—Parallel coordinate plot showing results of 1000 trials for hyperparameter optimization using the optuna package

Supplementary file3 (PNG 653 KB)—Tree plot showing a decision tree

Supplementary file4 (PNG 5140 KB)—Topfeatures (predictors) revealed by feature importance (Figure S3A), permutation importance (Figure S3B), and SHAP values (Figure S3C). Features in the plot are ordered by their relevance. The length of the bar corresponds to the feature’s importance

Supplementary file5 (DOCX 20 KB)

## Data Availability

Available upon reasonable request from the corresponding author.
