# Supplementary figures and images for: Development and application of explainable artificial intelligence using machine learning classification for long-term facial nerve function after vestibular schwannoma surgery
Source: J Neurooncol. 2024 Oct 11;171(1):165–77. doi: 10.1007/s11060-024-04844-7 (PMC11685252; doi:10.1007/s11060-024-04844-7)

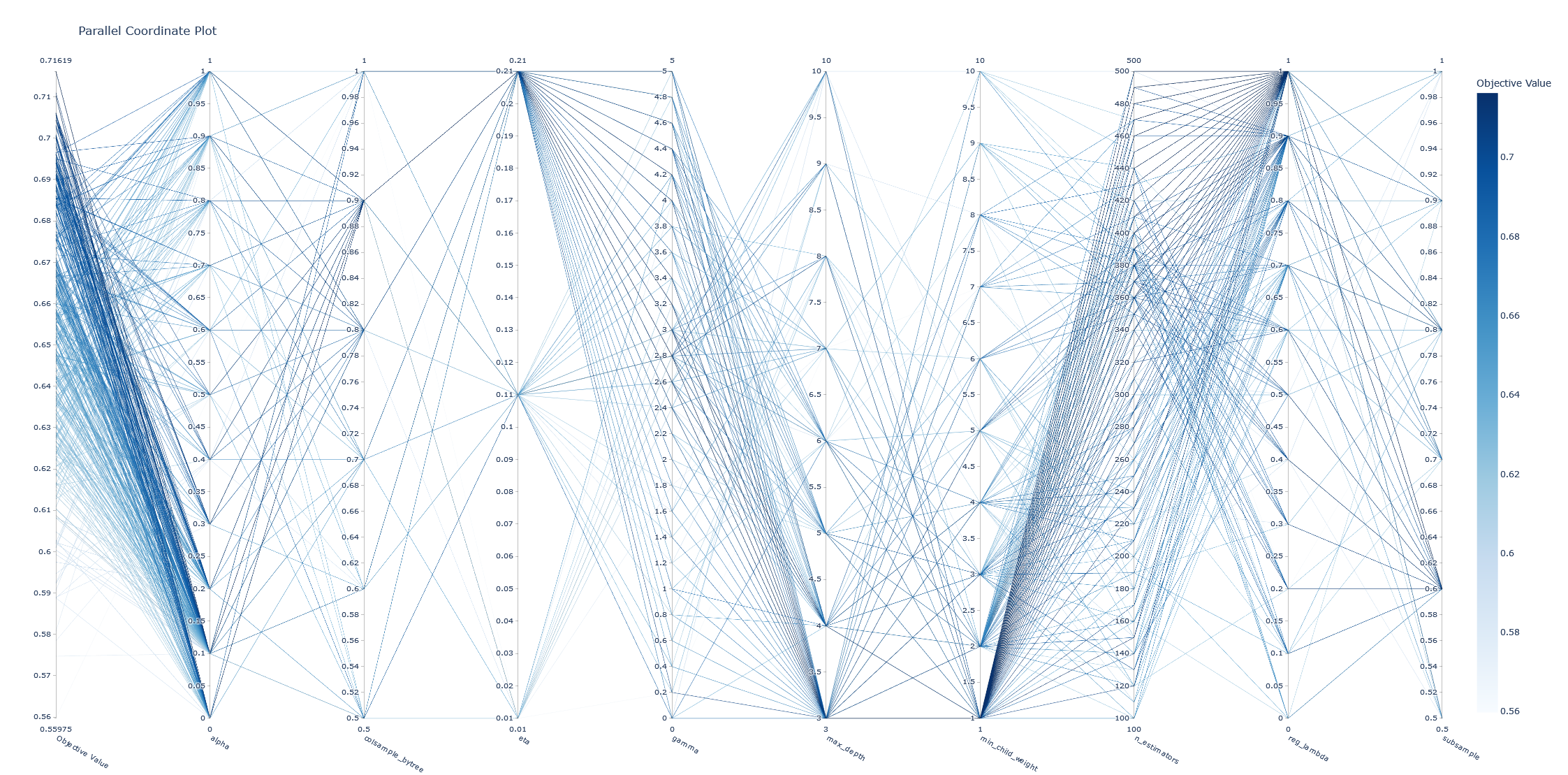

Supplement: Supplementary file 2 — Supplementary file2 (PNG 2152 KB)—Parallel coordinate plot showing results of 1000 trials for hyperparameter optimization using the optuna package [file 11060_2024_4844_MOESM2_ESM.png]

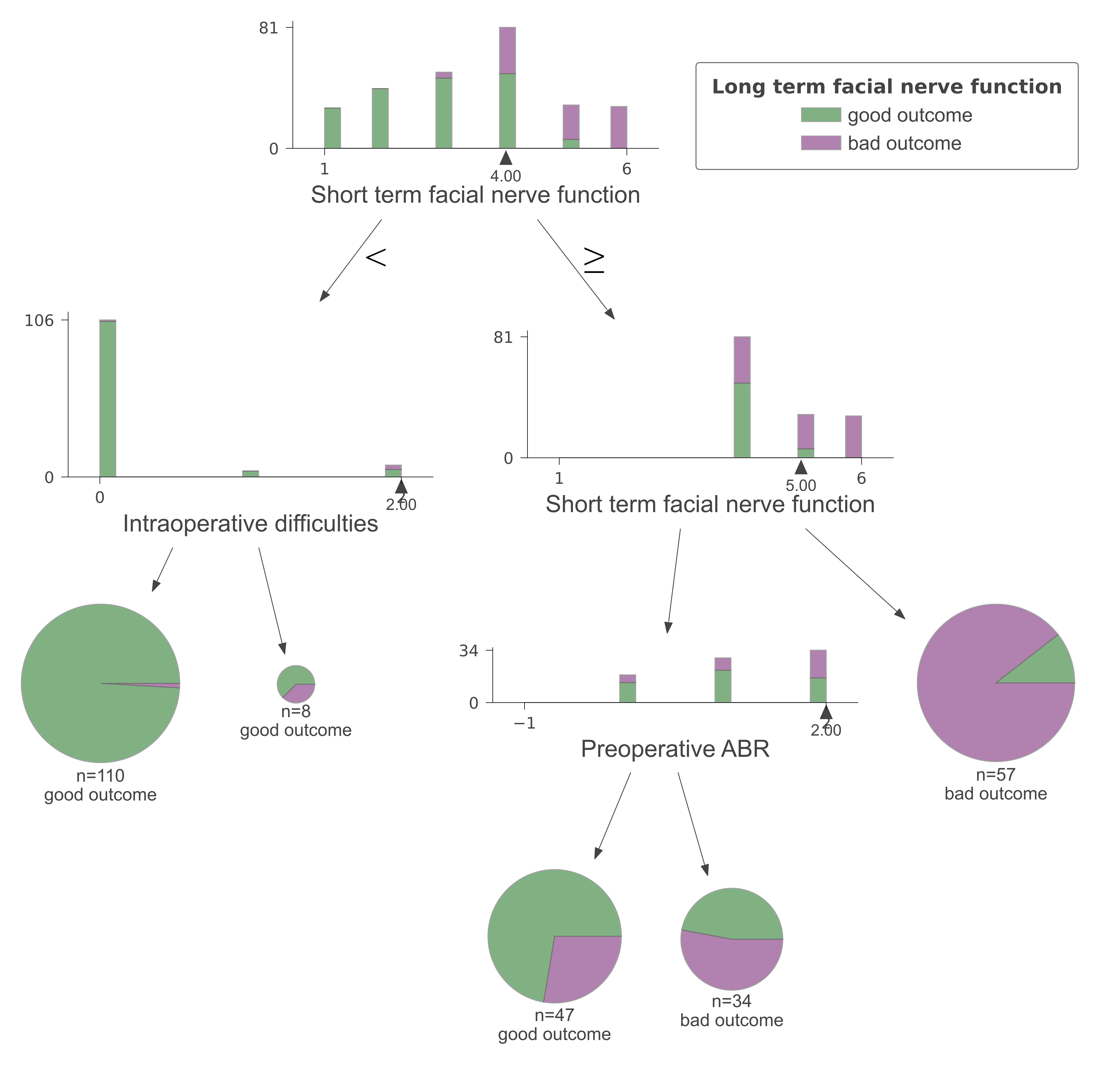

Supplement: Supplementary file 3 — Supplementary file3 (PNG 653 KB)—Tree plot showing a decision tree [file 11060_2024_4844_MOESM3_ESM.png]
