# Supplementary material for: Development and application of explainable artificial intelligence using machine learning classification for long-term facial nerve function after vestibular schwannoma surgery
Source: J Neurooncol. 2024 Oct 11;171(1):165–77. doi: 10.1007/s11060-024-04844-7 (PMC11685252; doi:10.1007/s11060-024-04844-7)
Supplement: Supplementary file 5 — Supplementary file5 (DOCX 20 KB) [file 11060_2024_4844_MOESM5_ESM.docx]

**Supplementary tables**

**Table S1**. Data description

| **Feature** | **type of feature (c-category, n-numeric)** | **Values** | **Encoded values** |
| --- | --- | --- | --- |
| Age during the surgery years | n | range: 18-74 | NA |
| Sex | c | female; male | 0; 1 |
| First symptom | c | incidental; deafness; hearing loss; tinnitus; ear pain; dizziness; headache; trigeminal nerve signs; facial nerve paresis; trigeminal neuralgia; cerebellar signs; papilledema | -1; 0; 1; 2; 3; 4; 5; 6; 7; 8; 9; 10 |
| Reason for the diagnosis | c | incidental; deafness; hearing loss; tinnitus; dizziness; headache; trigeminal nerve signs; facial nerve paresis; lower cranial nerve paresis; cerebellar signs; visual symptoms | -1, 0; 1; 2; 3; 4; 5; 6; 7; 8; 9 |
| Tinnitus | c | no; yes | 0; 1 |
| Hearing loss | c | no; medium; severe | 0; 1; 2 |
| Dizziness | c | no; yes | 0; 1 |
| Cerebellar signs | c | no; yes | 0; 1 |
| Trigeminal nerve signs | c | no; yes | 0; 1 |
| Preoperative headache | c | no; yes | 0; 1 |
| Preoperative hydrocephalus | c | no; yes | 0; 1 |
| Any previous treatment | c | no; yes | 0; 1 |
| Duration of symptoms months | c | range: 0-360 | NA |
| Preoperative ABR | c | Correct; not tested; other; abnormal; no response | -1; 0; 1; 2 |
| AAO HNS hearing classification | c | no data; A; B; C; D | 0; 1; 2; 3; 4 |
| Preoperative Samii hearing scale | n | range: 1-4 | NA |
| Preoperative House Brackmann scale | n | range: 1-6 | NA |
| Tumor volume mm3 | n | range: 0.2-113 | NA |
| Side of the tumor | c | left; right | 0; 1 |
| Internal auditory canal widening | c | normal; widened | 0; 1 |
| Surgeon | c | surgeon1; surgeon2; surgeon3; surgeon4 | 0; 1; 2; 3 |
| Surgical approach retrosigmoid | c | no; yes | 0; 1 |
| Surgical approach translabyrinthine and retrosigmoid | c | no; yes | 0; 1 |
| Bony opening | c | craniectomy; craniotomy | 0; 1 |
| Tumor structure | c | cystic; solid | 0; 1 |
| Surgical position | c | lateral decubitus; supine with head turned | 0; 1 |
| Neuromonitoring | c | no; yes | 0; 1 |
| Facial nerve dislocation pattern | c | anteriorly; no data; anterolaterally; anteromedially; inside tumor; posteriorly | 0; 1; 2; 3; 4; 5 |
| Intraoperative use of nimodipine | c | no; yes | 0; 1 |
| Intraoperative difficulties | c | none; adhesions; excessive bleeding | 0; 1; 2 |
| Histopathology | c | Antoni A; Antoni B; Antoni A and B | 0; 1; 2 |
| Preserved hearing after the surgery | c | hearing loss; deafness | 0; 1 |
| Postoperative complications | c | no; yes | 0; 1 |
| Short term facial nerve function | n | range: 1-6 | NA |
| Long term facial nerve function | c | bad outcome; good outcome | 0; 1 |

**Table S2.** Model comparisons. The best score obtained on five-fold cross-validation is bolded.

| **model** | **accuracy** | **roc_auc** | **MCC** | **f1_score** | **precision** | **recall** |
| --- | --- | --- | --- | --- | --- | --- |
| XGBoost | **0.86** | 0.91 | 0.69 | **0.80** | 0.80 | **0.80** |
| Logistic Regression | **0.86** | **0.93** | **0.69** | 0.78 | 0.81 | 0.78 |
| Decision Tree | 0.80 | 0.78 | 0.56 | 0.70 | 0.72 | 0.70 |
| Random Forest | 0.85 | 0.91 | 0.66 | 0.75 | **0.85** | 0.69 |

**Table S3**. Features (predictors) of depth 0 and interaction of depth 1. Information on feature self-interaction was removed

| **Interaction** | **Gain** | **wFScore** | **Average wFScore** | **Average Gain** | **Expected Gain** |
| --- | --- | --- | --- | --- | --- |
| Intraoperative difficulties\|Short term facial nerve function | 92.7 | 3.2 | 0.6 | 18.5 | 43.9 |
| Preoperative ABR\|Short term facial nerve function | 32.1 | 0.7 | 0.3 | 16.0 | 10.4 |
| Facial nerve dislocation pattern\|Short term facial nerve function | 25.1 | 1.0 | 0.5 | 12.6 | 12.5 |
| Preoperative ABR\|Preoperative Samii hearing scale | 23.9 | 0.7 | 0.7 | 23.9 | 17.6 |
| Postoeprative complications\|Short term facial nerve function | 23.5 | 0.4 | 0.4 | 23.5 | 8.9 |
| Preoperative Samii hearing scale\|Reason for the diagnosis | 20.4 | 0.3 | 0.3 | 20.4 | 5.4 |
| Preoperative Samii hearing scale\|Short term facial nerve function | 20.4 | 0.8 | 0.8 | 20.4 | 16.8 |
| Age during the surgery years\|Year of surgery | 16.4 | 1.6 | 0.8 | 8.2 | 13.3 |
| Age during the surgery years\|Preoperative ABR | 16.3 | 0.6 | 0.6 | 16.3 | 9.5 |
| Short term facial nerve function | 207.0 | 18.3 | 0.8 | 9.4 | 185.1 |
| Intraoperative difficulties | 39.1 | 5.0 | 0.6 | 4.9 | 24.7 |
| Age during the surgery years | 38.0 | 5.2 | 0.6 | 4.2 | 21.5 |
| Tumor volume mm3 | 34.3 | 8.1 | 0.7 | 3.1 | 23.0 |
| Preoperative Samii hearing scale | 25.2 | 2.8 | 0.9 | 8.4 | 24.1 |
| Facial nerve dislocation pattern | 24.3 | 3.0 | 0.5 | 4.0 | 12.5 |
| Preoperative ABR | 20.3 | 1.4 | 0.5 | 6.8 | 9.7 |
| First symptom | 17.3 | 3.3 | 0.8 | 4.3 | 14.2 |
| Year of surgery | 15.3 | 2.7 | 0.7 | 3.8 | 10.6 |
